# Effect of intracanal time of triple antibiotic paste on its removal from simulated immature roots using passive ultrasonic irrigation and XP-endo Finisher

**DOI:** 10.15171/joddd.2018.045

**Published:** 2018-12-19

**Authors:** Cangul Keskin, Duygu Hazal Güler, Evren Sarıyılmaz

**Affiliations:** ^1^Department of Endodontics, Faculty of Dentistry, Ondokuz Mayis University, Samsun, Turkey; ^2^Department of Endodontics, Faculty of Dentistry, Ordu University, Ordu, Turkey

**Keywords:** Irrigation, passive ultrasonic irrigation, triple antibiotic paste, XP-endo Finisher

## Abstract

***Background.*** The aim of this study was to evaluate the effectiveness of different irrigation techniques in the removal of triple antibiotic paste (TAP), which was applied for 7, 21 or 90 days, from simulated root canals of immature teeth.

***Methods.*** The root canal spaces of 190 maxillary canine teeth were filled with TAP and randomly divided into a control and 3 experimental groups according to the intracanal medicament period (7, 21 or 90 days). Syringe irrigation (SI), passive ultrasonic irrigation (PUI) and XP-endo Finisher (XP) were used for the removal of TAP (n=20). The amount of remaining medicament was calculated under a stereomicroscope using a 4-grade scoring system. Kruskal-Wallis H and Wilcoxon signedrank tests were used for statistical analyses (P<0.05).

***Results.*** The mean percentage of residual TAP was significantly greater in the SI group compared to PUI and XP at all the time intervals (P<0.05). Paste removal efficacy of PUI was not affected by the intracanal time of TAP (P>0.05), whereas the efficacy of SI and XP was significantly affected (P<0.05). No significant differences were detected between PUI and XP at 7- and 21-day intervals (P>0.05); however, at 90-day interval, PUI removed significantly greater amount of TAP than XP did (P<0.05).

***Conclusion.*** The time of the TAP in the root canal negatively affected the removal efficacy of the SI and XP-Endo Finisher; however, it did not affect the efficacy of the PUI.

## Introduction

Triple antibiotic paste (TAP), which was developed by Hoshino et al, is a mixture of metronidazole, ciprofloxacin and minocycline. It has been used to disinfect the root canal system in regenerative and conventional nonsurgical endodontic treatments.^1^ In regenerative pulp treatment, TAP is placed in the root canal system for several weeks so that its antimicrobial properties can create a suitable microenvironment for stem cell survival, proliferation and differentiation. Several case reports have reported the efficacy of the use of TAP in cases not responding to the use of calcium hydroxide.^1-3^ Since the minocycline within the TAP has been associated with tooth discoloration, alternative antibiotics such as cefaclor, fosfomycin and amoxicillin have been suggested as replacements for minocycline.^4-6^

The removal of TAP from the root canal system is another concern because TAP has been shown to have a detrimental effect on the human stem cells of apical papilla.^7^ Moreover, an animal study reported moderate inflammatory reactions in the subcutaneous tissues of rats.^8^ The presence of residual intracanal paste might also interfere with the properties of the sealers, such as setting, adaptation and penetration. Syringe irrigation was reported as inadequate for removing TAP from the root canal system completely.^9^ Numerous studies have investigated the efficacy of various irrigation solutions and activation techniques, emphasizing the difficulty of complete removal of intracanal medicaments.^9-11^ The XP-endo Finisher has been recently introduced as a unique instrument for enhanced final cleaning of root canal systems due to its increased flexibility and its ability to expand to adapt to the root canal in three dimensions.^12^ The effectiveness of the XP-endo Finisher in removing intracanal calcium hydroxide medicament from root canal systems has been reported in previous studies.^13^

The intracanal time of TAP varies according to the selected treatment procedure. A treatment period of three months before nonsurgical root canal treatment has been reported in several case reports, whereas applications for 1–3 weeks were considered sufficient before regenerative endodontic procedures.^2,14,15^ The American Association of Endodontics recommends an intracanal treatment period of 1–4 weeks for regenerative endodontic treatment and suggests additional treatment time if there are signs/symptoms of persistent infection.^16^ In vitro studies on the removal of TAP or on doubling antibiotic paste utilization as an intracanal medicament suggest periods varying between 1 and 4 weeks.^9,10,17^ To the best of the authors’ knowledge, there is no data yet on the effect of the intracanal time of TAP on the removal efficacy. The aim of this study was to evaluate the efficacy of syringe irrigation (SI), passive ultrasonic irrigation (PUI) and the XP-endo Finisher (XP) in removing TAP, which was applied for 7, 21 and 90 days in simulated immature root canal systems. The null hypothesis was that the removal of TAP was not affected by the irrigation technique or the intracanal medicament time.

## Methods

The local university ethics committee board approved the study protocol. A total of 190 freshly extracted human maxillary canine teeth with a single straight root and a patent single root canal were selected. Teeth with resorption, immature apices, previous endodontic treatment, cracks and fractures were excluded. Specimens were stored at 37°C and 100% humidity until the experiments. The apical 3 mm of the roots were resected to simulate an immature apex and then the crown surfaces were flattened to standardize the working length (WL) to 15 mm by using sterile diamond burs under water-cooling for each tooth. Endodontic access cavities were prepared by using sterile diamond burs and then the pulp tissue was extirpated using a #15 H-file (Dentsply Maillefer, Ballaigues, Switzerland). The root canals were prepared using ProTaper Next (Maillefer, Switzerland) instruments up to X5 (50.06) under copious irrigation with 1.5% NaOCl solution delivered with a 30-g needle (NaviTip; Ultradent, South Jordan, UT, USA). Following the chemo mechanical root canal preparation, the root canals were flushed with 2 mL of distilled water, 2.5 mL of 17% EDTA solution and 2 mL of distilled water, respectively. The root canals were dried with paper points.

The test apparatus was prepared as described by Topçuoğlu et al.^18^ The specimens were embedded in silicone impression material (Zetaplus, Zhermack, Rovigo, Italy) and placed in 1.5-mL Eppendorf tubes. Following the setting of the silicone, the specimens were removed and longitudinal grooves were prepared along the roots on the buccal and lingual surfaces. The specimens were carefully split into two halves using a hammer and chisel. Then a toothbrush was used to remove debris from the surfaces of root halves under running tap water. The root halves were dried and temporarily affixed using a small amount of cyanoacrylate glue (Scotch Super Glue gel; 3M, St Paul, MN, USA) to give them enough structural integrity to withstand the forces of the TAP placement and removal procedures until the microscopic observations could be carried out. The specimens were remounted in the impression material in the Eppendorf tubes. Five specimens were randomly selected as negative control and received no further procedures. TAP was prepared by mixing ciprofloxacin (BioFarma, Istanbul, Turkey), metronidazole (Sanofi, Istanbul, Turkey) and cefaclor (Basel, Istanbul, Turkey) powders in equal portions. Distilled water was added to the antibiotic powder mix until a creamy consistency was achieved. The freshly prepared TAP was applied to the root canals using a lentulo spiral until the paste was visible at the apical surface. Access cavities were sealed using a temporary sealing material (Cavit G, 3M ESPE, Seefeld, Germany). Five specimens were randomly selected as positive control and received no further procedure. The remaining 180 specimens were randomly divided into three groups (Groups 1, 2 and 3) according to the intracanal medicament period of 7, 21 and 90 days, respectively. The specimens in each group were assigned to 3 subgroups in terms of the removal procedure as described below (n=20):

**Groups 1:** The intracanal medicament was kept inside the canal for 7 days. After 7 days, the temporary sealing material was removed and TAP was removed as described below:

**Group 1–SI:** The temporary sealing material was removed and the root canals were flushed with 5 mL of 5.25% NaOCl for 1 minute followed by 1 minute of irrigation with 5 mL of 17% EDTA using a 30-g irrigation needle (Ultradent, USA) placed 1 mm short of the WL.

**Group 1–PUI:** The root canals were flushed with 5 mL of 5.25% NaOCl, which was ultrasonically activated with a 15.02 ultrasonic tip (ESI Instrument, EMS, Le Sentier, Switzerland) mounted on a piezoelectric ultrasonic unit (EMS) with the power setting at 6. The tip was placed 1 mm short of the WL and activated for 1 minute. The root canals were flushed again with 5 mL of 17% EDTA, which was activated for the following 1 minute. Each ultrasonic tip was used for three specimens and then discarded.

**Group 1–XP:** The root canals were irrigated with 5 mL of 5.25% NaOCl. XP-endo Finisher file was mounted in a torque controlled endodontic motor (VDW Gold, Munich, Germany) and cooled down (Chloraethyl, Dr. Georg Henning GmbH, Germany). Then the file was removed from its plastic tube with a slight lateral movement and operated according to manufacturer’s instructions with 800 rpm speed and 1 N.cm torque values for 1 minute with vertical movements of 7-8 mm to the full WL. Then the root canals were flushed with 5 mL of 17% EDTA solution and instrumented with XP-endo Finisher for another 1 minute. One XP-endo Finisher file per specimen was used.

**Groups 2:** The intracanal medicament was kept inside the canal for 90 days. After 90 days, the temporary sealing material was removed and TAP was removed in groups 2–SI, –PUI and –XP as described for groups 1–SI, –PUI and –XP, respectively.

**Groups 3:** The intracanal medicament was kept inside the canal for 90 days. After 90 days, the temporary sealing material was removed and TAP was removed in groups 3–SI, –PUI and –XP as described previously.

One experienced endodontist (C.K.) performed all the procedures described above. The temporarily glued halves were separated and each root half was visualized at ×30 magnification under a stereomicroscope. Two digital images were taken of each specimen. Two calibrated endodontists, who were blinded to the experimental groups, scored each image according to the classification described by van der Sluis et al.^19^

**Score 0:** The root canal was free of debris.

**Score 1:** Less than half of the root canal was filled with debris.

**Score 2:** More than half of the root canal was filled with debris.

**Score 3:** The root canal was completely filled with debris.

Based on the results of the Shapiro-Wilk test, non-parametric tests were performed.

Kruskal-Wallis and Wilcoxon signed-rank tests were used to analyze the differences in TAP removal scores of the study groups at selected time intervals. The level of significance was set at 0.05 and all the statistical analyses were performed using SPSS 21.0 (SPSS Inc., Chicago, IL, USA).

## Results

Interexaminer agreement was 97.4% as determined by a Kappa test. Table 1 details the distribution of the TAP removal scores for all the groups at all the time intervals. The positive control specimens confirmed that no intracanal antibiotic paste was removed during the disassembly and transportation processes. The scores of positive and negative control groups were significantly different from all the tested groups (P<0.05).

**Table 1:** Distribution of the TAP removal scores for all groups at all the time intervals

	**Paste removal scores**	**SI**	**PUI**	**XP**
**Group 1 (7 days)**	0	-	20 (50%)	19 (47.5%)
	1	23 (57.5%)	18 (45%)	15 (52.5%)
	2	13 (32.5%)	2 (5%)	-
	3	4 (10%)	-	-
**Group 2 (21 days)**	0	-	17 (42.5%)	11 (27.5%)
	1	14 (35%)	22 (55%)	28 (70%)
	2	23 (57.5%)	1 (5%)	1 (2.5%)
	3	3 (7.5%)	-	-
**Group 3 (90 days)**	0	-	18 (45%)	8 (20%)
	1	6 (15%)	22 (55%)	32 (80%)
	2	29 (72.5%)	-	-
	3	5 (12.5%)	-	-
**n (N)**		20(40)	20(40)	20(40)

In the SI groups, the 90-day group showed significantly higher scores compared to the 7-day and 21-day intervals (P<0.05). No significant difference was detected between the 7-day and 21-day intervals (P>0.05).

The paste removal efficacy of PUI was not affected by the intracanal time of the paste (P>0.05).

In the XP groups, the 7-day interval was associated with significantly less antibiotic paste than the 21-day and 90-day intervals (P<0.05). The difference between the 21-day and 90-day intervals was not significant (P>0.05).

PUI and the XP removed significantly more antibiotic paste than SI at all the time intervals (P<0.05). At the 7-day and 21-day intervals, no significant differences were detected between PUI and the XP; however, PUI removed significantly more antibiotic paste than the XP did after 90 days (P<0.05).

## Discussion

Several studies have evaluated the removal of different antibiotic pastes, including triple, double or modified triple antibiotic pastes and reported that no available technique or irrigation solution could completely remove the antibiotic paste from root canal systems.^9-11^ These studies used variable intracanal dressing periods ranging from 1 to 4 weeks; however, according to our literature search, there are no studies available on the effect of intracanal dressing duration on the removal of antibiotic paste. According to the results of this study, SI resulted in the worst scores at all the time intervals. At the 7-day and 21-day time intervals, PUI and XP were superior to SI in removing TAP, which was in accordance with the previous literature. Therefore, the null hypothesis was refuted.

Following the disinfection process, the antibiotic paste should be completely removed from the root canal to avoid any possible adverse interaction on the properties of the sealer and the root dentin.^20^ In revascularization procedures, removal of the paste is also important since it exerts detrimental effects on apical papilla stem cells.^7^ In addition, recent studies have shown that antibiotic paste reduces dentin microhardness and changes the chemical structure of dentin.^21,22^ The intracanal duration of polyantibiotic paste is dependent on the time required to eliminate the symptoms of infection. Endodontic infections are polymicrobial infections, in which both aerobic and anaerobic bacterial species are involved.^2,3^ TAP has been reported to be effective as an intracanal medicament for the treatment of teeth with large periapical lesions and persistent symptoms.^3^ Er et al reported that a large periapical lesion associated with a mandibular premolar tooth showed reduction and healing following TAP dressing for three months.^2^ The present study was the first to evaluate the removal of TAP kept in root canals for three months. The null hypothesis that the removal efficacy of TAP would not be affected by intracanal duration was also refuted. The results of this study showed that the removal efficacy of PUI was not affected by the intracanal duration of TAP, whereas the efficacy of SI and the XP in removing TAP from root canals was time-dependent.

The efficacy of PUI in removing intracanal medicaments and antibiotic paste has been reported in the literature.^13,17,23^ A recent systematic review stated that there is sufficient evidence regarding the efficacy of ultrasonically activated irrigation.^23^ PUI creates cavitation and microstreaming effects and contributes to the removal of intracanal TAP by streaming in an apical-to-coronal direction.^19^ In the present study, the velocity created within the solution by PUI removed the TAP regardless of the intracanal duration.

In the present study, the intracanal period of TAP significantly affected the efficacy of the XP-endo Finisher. In the 7-day group, the XP-endo Finisher completely removed TAP from 47.5% of the specimens, whereas in the 21-day and 90-day groups, this rate decreased to 27.5% and 20%, respectively. Minocycline within the conventional TAP binds to calcium ions via chelation and forms an insoluble complex, which has been associated with severe tooth discoloration.^24,25^ Berkhoff et al reported that the removal of the conventional TAP was much more difficult compared with the removal of calcium hydroxide.^9^ To overcome these problems, the use of alternative antibiotics to replace minocycline in the combination has been suggested.^4,26,27^ Therefore, in the present study, TAP was modified by replacing minocycline with cefaclor, which was suggested as an alternative due to its antimicrobial properties within the root canal system.^5^ Antibiotic paste has been reported to be an acidic material, which might exert demineralizing effects on the root dentin.^21,22^ Any possible interaction between the modified TAP containing cefaclor and the root dentin in prolonged use warrants future investigation.

A simulated immature root model, which provides standardization of the dimensions of root canal spaces, was used in this study. However, the inability to detect the paste diffused into dentinal tubules and the lack of simulation of the complexity of the root canal system present limitations to this model.

## Conclusions

Within the limitations of this in vitro study, the intracanal time of TAP negatively affected the removal efficacy of the XP-endo Finisher and SI. Nevertheless, the efficacy of PUI in removing TAP was not influenced by the intracanal time of the paste.

## Authors’ contributions

Please add authors’ contributions according to the Journal format.

## Acknowledgments

Authors deny any conflict of interest.

## Funding

Please add funding statement.

## Competing interests

Please add a statement for competing interests.

## Ethics approval

Please add ethics clearance statement or code.
